# Attacking and goal-scoring trends among top teams in EHF EURO handball (2016–2024): implications for representative practice design

**DOI:** 10.3389/fspor.2026.1771752

**Published:** 2026-03-16

**Authors:** Truls Valland Roaas, Håvard Lorås

**Affiliations:** 1 Department of Sport Sciences, Faculty of Education and Arts, Nord University, Levanger, Norway; 2 Department of Teacher Education, Faculty of Social and Educational Sciences, NTNU, Trondheim, Norway

**Keywords:** decisive variables, ecological dynamics, handball coaching, match trends, performance development

## Abstract

**Introduction:**

This study examined longitudinal trends in attacking play and goal-scoring patterns among top-performing men’s and women’s teams in elite international handball.

**Methods:**

Match performance data were analysed from 666 matches across 10 European Handball Federation EURO Championships (five men’s and five women’s tournaments) conducted between 2016 and 2024.

**Results:**

The findings revealed a consistent increase in the contribution of positional attacks to goal scoring across both men’s and women’s competitions. This was accompanied by marked increases in 6-m finishes and breakthrough goals. In contrast, fast-break scoring remained stable or showed a slight decline over time, suggesting a growing emphasis on structured offensive play against increasingly organised defensive systems. Spatial analyses indicated a progressive centralisation of scoring activity towards close-range zones, while wing and 9-m shooting exhibited more variable or declining patterns across tournaments. Gender-based comparisons indicated that men produced higher outputs in several structured attack categories, whereas no meaningful gender differences were observed in transitional or numerical imbalance situations.

**Discussion:**

These findings indicate a systematic shift in the offensive ecology of elite championship handball towards centrally organised, high-probability scoring actions. Implications are discussed through the lens of representative learning design, highlighting how empirically derived match patterns can inform the design of representative practice tasks that preserve key perceptual–temporal constraints of competition. By aligning training environments with stable performance regularities observed at the highest level, coaches can better support functional decision-making and transfer from training to match play within comparable elite contexts.

## Introduction

Team handball is among the most tactically intricate and physically demanding invasion sports, necessitating the seamless integration of physiological capacity, technical execution, tactical intelligence, and psychological resilience (1, 2). Originating in Northern Europe in the late 19th century, the sport has evolved into a globally recognised discipline. Nevertheless, its competitive epicentre remains firmly rooted in Europe, where elite leagues and national programs maintain exceptionally high standards (3). Played on a 40 × 20-m court by two teams of seven players across two 30-min halves, handball is characterised by high-intensity transitions between offence and defence, frequent high-speed sprints, explosive jumps, and throws potentially exceeding 100 km/h (4, 5). At the highest level, players are distinguished not only by physical and technical superiority but also by their ability to synthesise anthropometric advantages, role-specific expertise, advanced recovery strategies, and quick decision-making within coordinated team synergies. This expertise encompasses anticipatory defensive behaviour, offensive behaviour, accurate passing under pressure, and a diverse shooting repertoire (6–8). Concurrently, tactical success depends on the integration of structured collective coordination with real-time adaptive responses to opponent strategies (9).

Attacking play and offensive actions in team handball are intrinsically multidimensional, requiring the convergence of physical effort, technical skill, and tactical awareness to create and exploit scoring opportunities. Offensive efficiency depends heavily on fluid transitions from defence to attack, rapid spatial exploitation, and the execution of tactical frameworks that destabilise organised defensive systems. Central offensive behaviours include dynamic ball circulation, synchronised player movement, and strategic positional rotations, all of which significantly contribute to attacking success (10). High-performing teams tend to favour well-orchestrated offensive patterns—such as fast transitions, structured counterattacks, and elaborate positional plays—over improvisational and disjointed actions (11). Regulatory changes and technological innovations over the past decade have shaped new offensive possibilities and adaptations in elite team handball. For instance, the implementation of the empty-goal rule, which allows for the temporary substitution of the goalkeeper with a field player, has expanded tactical variability and necessitated new decision-making strategies under pressure (12). Similarly, amendments to passive-play regulations have increased the overall pace of the game, requiring faster, more decisive offensive executions (13). On the technological front, advances within video analytics, GPS tracking, and AI-driven performance modelling have transformed coaching methodologies, enabling detailed assessments of passing networks, shot efficiency, and spatial utilisation patterns (9, 14–16).

Within this evolving tactical ecology, positional attacks initiated against organised defences have been described as increasingly significant in recent performance analyses (11, 17, 18). These structured offensive attacks rely on precisely timed passes, spatial manipulation, and rehearsed tactical movements to disrupt defensive cohesion and open scoring lanes (19). In contrast, fast breaks are considered the most effective scoring method, leveraging defensive disorganisation to generate high-quality open opportunities through speed, deception, and rapid decision-making (10). Offensive behaviours are also position-dependent: Backcourt players specialise in long-range shooting and breakthrough penetrations; wing players exploit angular space for jump shots; and pivot players operate in high-contact zones near the 6-m line, facilitating close-range finishes. Indeed, recent data on elite team handball have reported patterns consistent with increased 1-on-1 duels in centralised attacking zones, particularly involving pivot and breakthrough play, indicative of broader evolutions in offensive coordination (11).

To fully evaluate offensive effectiveness, a multidimensional analytic lens is required. Contemporary performance frameworks increasingly advocate for integrative efficiency indices that account for players' holistic contributions across complex offensive sequences (17). However, traditional metrics—such as shot success rate, goal spatial distribution, turnover frequency, and conversion rate—remain essential. Accordingly, coaches now leverage advanced data analytics to discern emergent team patterns, refine tactical structures, and inform evidence-based training interventions.

Against this backdrop, limitations of traditional skill acquisition paradigms—often characterised by reductionist, decontextualised, and overly prescriptive training formats—have become increasingly evident in team sports (20). Such approaches frequently dissociate practice from the informational and temporal demands of match play, thereby constraining the ability of athletes to adapt actions to the dynamic conditions encountered in competition. As an alternative framework, Representative Learning Design (RLD) has emerged from ecological dynamics as a principled approach for aligning practice environments with the functional constraints of performance (21). Rooted in an ecological perspective on skill acquisition (22, 23), RLD emphasises that skilled behaviour emerges through continuous interaction between performers and their environment, rather than through the internalisation of idealised movement patterns. Central to this framework is the notion of representativeness, which refers not to superficial realism, but to the preservation of key informational constraints that regulate perception–action coupling in competition (24). When practice tasks fail to maintain these constraints, athletes may attune to non-functional information, thereby limiting the transfer of learning to performance settings (25).

Within this framework, RLD is operationalised through the dual criteria of informational functionality and action fidelity (24, 26). Informational functionality concerns whether the perceptual variables available in practice correspond to those constraining decision-making and action in competition, while action fidelity refers to the extent to which behaviours elicited in practice resemble those required under competitive temporal and spatial pressures. Importantly, RLD does not prescribe specific training methods; rather, it provides a design logic through which coaches manipulate constraints to invite exploration, coordination, and the emergence of functional solutions (21, 27). From an applied perspective, the effectiveness of representative practice design depends on accurately identifying which informational constraints are most decisive for successful performance. In invasion sports such as team handball, these constraints are inherently embedded in recurring patterns of interaction between players, spatial configurations around key scoring zones, and the temporal organisation of attacking sequences. Consequently, empirical analyses of match performance are essential for specifying the task-relevant regularities that practice environments should preserve. Without such empirical grounding, representative practice risks being guided by normative assumptions rather than by the functional structure of the game itself.

In the present study, RLD is not treated as a testable learning mechanism, but rather as a lens through which match-derived regularities may inform the design of representative practice tasks. Understanding how goals are produced, where decisive actions occur, and which offensive configurations are most prevalent at the highest competitive level provides empirically grounded constraints that can guide practice design. From this perspective, longitudinal analyses of top elite match performance offer a critical foundation for aligning training environments with the evolving ecological demands of top-performing team handball. In particular, our dataset comprises 10 EHF EURO championships in total, consisting of five men's and five women's tournaments held in 2016, 2018, 2020, 2022, and 2024.

The overall aim of the current study was thus to examine the trends in attacking play and goal-scoring among top-performing teams in the European Handball Federation EURO championships (men's and women's) over the past decade and discuss the implications for representative practice designs targeted at the dynamics of offensive attacking. The following research questions thus guided this work:

What is the distribution and frequency of goals scored across different types of attacks in male and female top-performing handball teams across the 2016–2024 European championships?
What is the spatial distribution of goal-scoring in top-performing male and female handball teams across various on-court shooting positions from the 2016–2024 European championships?
What implications do the observed goal-scoring patterns have for the design of representative practice tasks in elite team handball?

## Materials and methods

To investigate top-performing representativeness in attacking play, we conducted a retrospective study design utilising secondary data comprising cumulative performance statistics from the top-performing teams in the European Handball Federation (EHF) EURO championships (men's and women's) over the past decade. European nations dominate international handball, with rare exceptions such as South Korea (women: 1988, 1992, 1995) and Brazil (women: 2013) winning the Olympic or World Championship titles. Given this dominance, the EHF EURO championships provide a highly competitive environment for analysis, minimising quality disparities between competing teams.

### Sample

The sample comprised match performance data from the European Handball Federation (EHF) EURO Handball Championships conducted between 2016 and 2024 at the senior international level. In total, 10 tournaments were included, corresponding to five men's and five women's championships held in 2016, 2018, 2020, 2022, and 2024.

To ensure comparability of competitive exposure and analytical relevance, the sample was restricted to top-performing teams, operationally defined as national teams progressing beyond the preliminary round of each championship. Teams were included if they played more than six matches (>6) within a given tournament. This threshold corresponds to participation beyond the initial group phase under the prevailing competition formats during the study period and ensured that all included teams had extended tournament involvement, typically reaching the main round and/or knockout stages. Thus, the sample reflects performance patterns among teams with sustained competitive exposure rather than exclusively medal-winning teams.

Due to structural changes in tournament formats across the study period, the number of teams meeting the inclusion criterion varied between championships. In the men's EHF EURO tournaments, 16 teams participated in 2016 and 2018, whereas 24 teams competed in the 2020, 2022, and 2024 editions. In the 2016 men's championship, additional placement matches for seventh position were played, resulting in two more teams exceeding the inclusion threshold compared with the 2018 tournament. On the women's side, the number of participating teams increased from 16 to 24 in the 2024 EHF EURO championship. Importantly, the same inclusion criterion (>6 matches played) was applied consistently across tournaments and genders, with observed variation in sample size reflecting changes in competition structure rather than alterations in sampling logic. As teams were only included in tournaments in which they met the inclusion criterion (>6 matches played), not all national teams contributed observations to all championships. Consequently, team participation varied across tournament years, and tournament-level match totals reflected both the number of included teams and the prevailing competition formats. All matches played by the included teams within each tournament were analysed. Match statistics were obtained from the official EHF data provider (Swiss Timing), which employs standardised event-coding protocols across competitions and years to ensure consistency in data collection.

For analytical purposes, the unit of observation was defined as team × tournament, such that each national team's performance within a given championship constituted one observational case. Several national teams appeared in multiple tournaments across the study period, contributing repeated observations. This longitudinal structure was explicitly accounted for in the statistical modelling (see Statistical Analysis).

The final dataset comprised match data from 36 female teams representing 14 nations and 50 male teams representing 18 nations across the 10 championships. Most national teams therefore appeared in more than one tournament. Included teams played between seven and nine matches per championship, resulting in a total of 278 matches in the women's tournaments and 388 matches in the men's tournaments. The distribution of teams and the number of matches played per team within each tournament are presented in Tables 1, 2.

**Table 1:** Teams and the number of matches included from each male EHF EURO championship.

Team/year	EURO 2016	EURO 2018	EURO 2020	EURO 2022	EURO 2024	Total matches played (across included tournaments)
Sweden	7	8	7	9	9	**40**
Croatia	8	7	9	7	7	**38**
Spain	8	8	9	9	(-)	**34**
Denmark	7	8	(-)	9	9	**33**
France	7	8	(-)	9	9	**33**
Germany	8	(-)	8	7	9	**32**
Norway	8	(-)	9	8	7	**32**
Iceland	(-)	(-)	7	8	7	**22**
Slovenia	(-)	(-)	9	(-)	8	**17**
Hungary	(-)	(-)	7	(-)	8	**15**
Portugal	(-)	(-)	8	(-)	7	**15**
CzechRep.	(-)	7	7	(-)	(-)	**14**
Austria	(-)	(-)	7	(-)	7	**14**
Poland	7	(-)	(-)	7	(-)	**14**
Netherlands	(-)	(-)	(-)	7	7	**14**
Belarus	(-)	(-)	7	(-)	(-)	**7**
Montenegro	(-)	(-)	(-)	7	(-)	**7**
Russia	(-)	(-)	(-)	7	(-)	**7**
**MP**	**60**	**46**	**94**	**94**	**94**	**388**

**MP**, Matches played for included teams. Cells marked with (-) indicate that the team did not meet the inclusion criterion (>6 matches played) in that tournament and was therefore not included for that championship. Total matches represent the sum of matches played by each team across all tournaments in which they met the inclusion criterion. Tournament totals reflect the number of included teams and matches played under the respective competition formats, which varied across championship years.

Bold values represent the total number of matches.

**Table 2:** Teams and the number of matches included from each female EHF EURO championship *.*

Team/year	EURO 2016	EURO 2018	EURO 2020	EURO 2022	EURO 2024	Total matches played (across included tournaments)
France	8	8	8	8	9	**41**
Norway	8	7	8	8	9	**40**
Netherlands	8	8	7	7	8	**38**
Denmark	8	(-)	8	8	9	**33**
Sweden	(-)	7	(-)	7	8	**22**
Romania	7	8	(-)	(-)	7	**22**
Russia	(-)	8	7	(-)	(-)	**15**
Montenegro	(-)	(-)	(-)	8	7	**15**
Germany	7	(-)	(-)	(-)	7	**14**
Hungary	(-)	(-)	(-)	(-)	9	**9**
Croatia	(-)	(-)	8	(-)	(-)	**8**
Poland	(-)	(-)	(-)	(-)	7	**7**
Switzerland	(-)	(-)	(-)	(-)	7	**7**
Slovenia	(-)	(-)	(-)	(-)	7	**7**
TOTAL MP	**46**	**46**	**46**	**46**	**94**	**278**

**MP**, Matches played for included teams. Cells marked with (-) indicate that the team did not meet the inclusion criterion (>6 matches played) in that tournament and was therefore not included for that championship. Total matches represent the sum of matches played by each team across all tournaments in which they met the inclusion criterion. Tournament totals reflect the number of included teams and matches played under the respective competition formats, which varied across championship years.

Bold values represent the total number of matches.

### Data collection and variables

All performance data were obtained from the official European Handball Federation (EHF) website and recorded using Swiss Timing technology. Offensive performance variables were defined and coded according to the standardised Swiss Timing operational framework. To ensure construct validity and transparency, all extracted variables, their exact Swiss Timing labels, operational definitions, and classification stability across tournaments are presented in Supplementary Table S1. According to the Swiss Timing documentation, no changes to the operational definitions of the variables analysed in the present study were introduced during the 2016–2024 EHF EURO period.

### Statistical analysis

All attacking and goal-scoring variables were divided by the total number of games played by each team in each tournament, to control for national team differences in matches played in each tournament. This procedure—with counts normalised by games—produces dependent variables that are bounded, non-normal, rate-like measures that cannot be considered continuous Gaussian outcomes. Furthermore, most national teams appear in multiple tournaments (see Tables 1, 2). The unit of analysis was thus team × tournament observations. As teams could contribute data across multiple tournaments, each statistical model included a random intercept for team to account for within-team dependence across repeated observations. Fixed effects included gender, tournament (treated as categorical), and their interaction. The primary hypotheses were evaluated via Type III tests of (a) the main effect of tournament, (b) the main effect of gender, and (c) the gender × tournament interaction. All dependent variables were modelled using a Gamma distribution with a log link to accommodate positive, right-skewed outcome distributions and yield multiplicative effects on the expected outcomes. Model estimation was performed using maximum likelihood. Model adequacy was evaluated using convergence diagnostics and information criteria (including the small-sample corrected Akaike Information Criterion) when comparing alternative specifications fit to the same dataset. For significant omnibus effects and interactions, estimated marginal means and pairwise contrasts were examined to aid interpretation, with 95% confidence intervals reported. Effect sizes for fixed effects were summarised using partial eta squared ($\eta_{p}^{2}$) computed from Wald *F* tests. These values were treated as approximate indices of explained variance given the mixed-model framework and non-normal outcome family. Effect sizes of approximately 0.01, 0.06, and 0.14 were interpreted as small, medium, and large effects, respectively (34, 35). All analyses were conducted in SPSS Predictive Analytics version 31.0 (IBM, Armonk, NY, United States) using generalised linear mixed models (GENLINMIXED), with alpha = 0.05 as the criterion for statistical significance. This modelling strategy accounts for the non-independence of repeated team observations across tournaments and allows robust estimation of longitudinal performance patterns within teams. As tournament formats changed over the study period (e.g., expansion of women's championships in 2024), tournament effects may partly reflect structural differences in competition format in addition to tactical evolution.

## Results

The five women's tournaments in the dataset contained a total of 15,285 attacks, resulting in 12,738 shots, and 6,662 goals. Across the five men's tournaments, 20,089 attacks were recorded, resulting in 17,862 shots, which translated into 11,146 goals. There were 1,087 (9.75%) and 817 (10.66%) 7-m penalty goals in total in the men's and women's tournaments, respectively. These set-piece goals were excluded from further analysis, as the current study focuses on attacking and scoring patterns. Descriptive statistics for type of goals scored per average match across male and female EHF European Championships are presented in Tables 3, 4, respectively. In Tables 3, 4, sample sizes (n) represent the number of teams included in each tournament, with each team contributing one tournament-level observation based on per-match averages.

**Table 3:** Descriptive statistics (mean, SD) for type of goals scored per match across male European championships.

Type of goal	Team handball European championship					
	2016 (*n* = 8)	2018 (*n* = 6)	2020 (*n* = 12)	2022 (*n* = 12)	2024 (*n* = 12)	Total (*n* = 50)
Positional attack	23.25 (1.23)	23.19 (1.58)	24.08 (1.94)	24.59 (1.59)	25.79 (1.70)	24.37 (1.85)
Majority attack	4.07 (0.85)	3.48 (0.99)	4.10 (0.93)	4.01 (0.67)	3.41 (0.93)	3.83 (0.89)
Minority attack	1.87 (0.52)	1.87 (0.30)	2.03 (0.42)	1.94 (0.59)	1.95 (0.48)	1.95 (0.47)
Nine-metre shot	8.31 (1.54)	8.14 (1.69)	7.21 (1.16)	5.53 (1.89)	6.31 (1.29)	6.88 (1.78)
Six-metre centre shot	4.59 (0.87)	3.85 (1.27)	5.39 (1.46)	6.62 (1.83)	8.82 (1.60)	6.20 (2.25)
Wing shot	5.32 (1.04)	5.41 (1.81)	5.87 (1.15)	5.63 (1.42)	3.77 (1.08)	5.16 (1.47)
Fast break	4.46 (1.49)	4.55 (1.32)	3.41 (0.92)	3.65 (0.83)	3.31 (0.87)	3.75 (1.12)
Team fast break	3.74 (1.01)	3.54 (0.78)	2.89 (0.69)	2.92 (0.71)	3.07 (0.85)	3.16 (0.83)
Individual fast break	0.71 (0.83)	1.01 (0.59)	0.52 (0.54)	0.72 (0.60)	0.24 (0.15)	0.59 (0.59)
Breakthrough	2.10 (1.36)	2.90 (1.43)	3.18 (1.19)	3.91 (1.73)	3.98 (0.88)	3.34 (1.45)
Fast throw-off	0.39 (0.42)	0.45 (0.36)	0.29 (0.21)	0.29 (0.36)	0.38 (0.25)	0.35 (0.31)

*n* refers to the number of national teams included in each championship. Each team contributes one observation per tournament, based on average values per match.

**Table 4:** Descriptive statistics (mean, SD) for type of goals scored per match across female European championships.

Type of goal	Team handball European championship					
	2016 (*n* = 6)	2018 (*n* = 6)	2020 (*n* = 6)	2022 (*n* = 6)	2024 (*n* = 12)	Total (*n* = 36)
Positional attack	20.85 (2.16)	23.64 (1.35)	23.59 (1.59)	24.32 (1.47)	23.81 (1.92)	23.34 (2.02)
Majority attack	3.16 (0.44)	3.68 (1.07)	3.67 (0.77)	3.15 (1.21)	3.64 (0.85)	3.49 (0.88)
Minority attack	1.87 (0.77)	1.67 (0.66)	1.64 (0.54)	2.29 (1.03)	1.96 (0.61)	1.90 (0.71)
Wing shot	4.10 (0.82)	6.81 (1.72)	7.48 (1.54)	7.40 (1.29)	4.62 (1.16)	5.84 (1.89)
Nine-metre shot	6.82 (1.01)	6.85 (0.79)	5.74 (1.06)	5.19 (1.05)	4.48 (1.24)	5.59 (1.43)
Six-metre centre shot	3.91 (1.50)	4.55 (1.36)	4.36 (1.05)	4.57 (0.99)	7.06 (1.27)	5.25 (1.77)
Breakthrough	2.95 (1.23)	2.11 (0.96)	3.24 (1.19)	4.27 (1.50)	4.88 (1.68)	3.72 (1.69)
Fast break	3.62 (1.44)	3.77 (1.50)	3.56 (1.65)	3.39 (1.55)	3.15 (1.77)	3.44 (1.55)
Team fast break	2.62 (1.15)	3.15 (1.46)	2.89 (1.38)	3.17 (1.48)	2.87 (1.57)	2.93 (1.37)
Individual fast break	0.99 (0.35)	0.62 (0.27)	0.66 (0.62)	0.22 (0.23)	0.28 (0.25)	0.51 (0.44)
Fast throw-off	0.55 (0.81)	0.26 (0.21)	0.06 (0.15)	0.17 (0.13)	0.38 (0.41)	0.30 (0.43)

*n* refers to the number of national teams included in each championship. Each team contributes one observation per tournament, based on average values per match.

### Goals from different types of attacks

**Figure 1:**
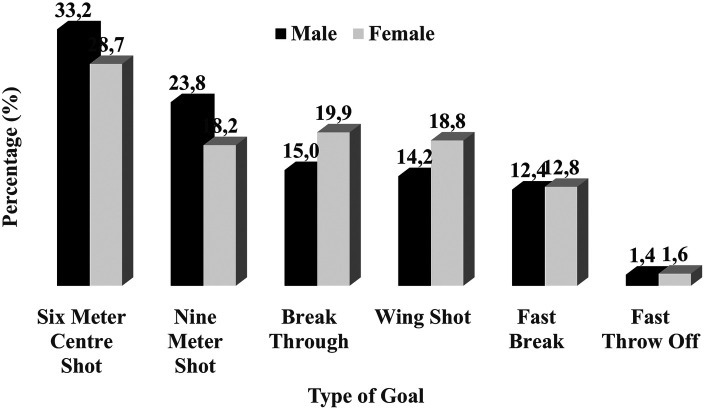
Frequency distribution of the attacking goals for men and women in the 2024 European championship.

In the 2024 tournament, the number of *goals from attacks* per average was 28.45 for men’s teams and 26.84 for women’s teams, respectively. There was a significant main effect of gender [*F* (1, 76) = 13.96, *p* < 0.001, $\eta_{p}^{2}-=0.16$*]*, but no significant effect of tournament (*p* = 0.43) or tournament × gender interaction (*p* = 0.16) on the number of goals scored from offensive attacks. Most of these goals originated from *positional attacks* (i.e., offensive sequences with the defensive team being fully organised), amounting to 90.6% for men's teams and 88.7% for women's teams in the 2024 tournament. As shown in Table 5 and Figure 1, there was a significant main effect of gender on the proportion of goals from *positional attacks* in an average match, with men’s teams showing significantly higher values than women’s teams [medium effect size, mean difference = 1.21 goals, 95% CI (0.49, 1.92)]. The generalised linear mixed model indicated no other significant effects of gender, EHF tournament, or gender × tournament interactions when fitted to goals scored from *positional attacks*, *majority attacks*, or *minority attacks* per average match. The two latter categories accounted for ≤13% of goals from attacks. Similarly, no significant main effects of gender, EHF Euro tournament, or gender × tournament interaction were indicated when GLMMs were fitted to *team/individual fast-break goals* (offensive transitions without defending team in full defensive organisation) or fast throw-offs (transition attack immediately after opponent scoring). However, there was a significant main effect of gender on total amount of *fast breaks*, with men’s teams showing significantly higher values than women’s teams [small effect size, mean difference = 0.52 goals, 95% CI (0.03, 1.03)]. *Fast-break goals* typically accounted for a small proportion of goals (≤13%) from attacks in an average match for both men’s and women's teams.

**Table 5:** Overview of results from generalised linear mixed models.

Type of goal	Gender ^[*F* (1,76)]^	EHF tournament ^[*F* (4, 76)]^	Gender×tournament ^[*F* (4, 76)]^
Positional attack	10.68, *p* = 0**.002**, $\eta_{p}^{2}=0.12$	1.28, *p* = 0.285, $\eta_{p}^{2}=0.06$	2.20, *p* = 0.077, $\eta_{p}^{2}=0.10$
Majority attack	3.08, *p* = 0.083, $\eta_{p}^{2}=0.04$	0.07, *p* = 0.990, $\eta_{p}^{2}<0.01$	1.68, *p* = 0.163, $\eta_{p}^{2}=0.08$
Minority attack	0.20, *p* = 0.654, $\eta_{p}^{2}<0.01$	0.13, *p* = 0.973, $\eta_{p}^{2}<0.01$	0.83, *p* = 0.508, $\eta_{p}^{2}-=0.04$
Wing shot	5.13, *p* = **0.026**, $\eta_{p}^{2}=0.06$	2.19, *p* = 0.077, $\eta_{p}^{2}=0.44$	3.65, *p* = 0**.009**, $\eta_{p}^{2}=0.16$
Nine-metre shot	15.24, *p* < **0.001**, $\eta_{p}^{2}=0.17$	1.50, *p* = 0.209, $\eta_{\;p6}^{2}=0.07$	0.97, *p* = 0.428, $\eta_{p}^{2}=0.05$
Six-metre centre shot	8.15, *p* = **0.006**, $\eta_{p}^{2}=0.10$	2.82, *p* = **0.031**, $\eta_{p}^{2}=0.13$	2.02, *p* = 0.100, $\eta_{p}^{2}=0.05$
Breakthrough	0.21, *p* = 0.650, $\eta_{p}^{2}<0.01$	0.84, *p* = 0.505, $\eta_{p}^{2}=0.04$	1.14, *p* = 0.346, $\eta_{p}^{2}<0.01$
Fast break			
Team fast break	4.50, *p* = **0.037**, $\eta_{p}^{2}=0.06$	0.14, *p* = 0.966, $\eta_{p}^{2}<0.01$	0.20, *p* = 0.938, $\eta_{p}^{2}=0.01$
Individual fast break	1.35, *p* = 0.249, $\eta_{p}^{2}=0.02$	0.72, *p* = 0.576, $\eta_{p}^{2}=0.04$	2.49, *p* = 0.050, $\eta_{p}^{2}=0.07$
Fast throw-off	1.40, *p* = 0.240, $\eta_{p}^{2}=0.02$	0.35, *p* = 0.841, $\eta_{p}^{2}=0.02$	0.94, *p* = 0.448, $\eta_{p}^{2}=0.05$

$\eta_{p}^{2}$, partial eta squared (effect size).

Bold values represent statistically significant differences.

### Spatial distribution of scored goals

As indicated in Tables 3, 4, the proportion of *breakthrough* goals (individual attacking actions in the 9-m zone) amounted to 4–5 goals in an average match. Analysis of these types of goals—when fitted to generalised linear mixed models—indicated no significant main effects of gender, EHF Euro tournament, or gender × tournament interaction. When considering goals scored from other on-court positions, however, a significant main effect of gender was observed for *wing shots* [small effect size, mean difference = 0.66 goals, 95% CI (0.07, 1.26)], *9-m shots* [large effect size, mean difference = 1.23 goals, 95% CI (0.62, 1.94)], and *6-m centre shots* [medium effect size, mean difference = 0.85 goals, 95% CI (0.25, 1.45)]. Female teams tended to have higher values for *wing shot* goals, and male teams for *9*- and *6-m* goals. Furthermore, a significant gender × tournament interaction effect (large) was observed for a proportion of *wing shots* in an average match. In 2022 [mean difference = 1.71 goals, 95% CI (0.11, 3.30)] and 2024 [mean difference = 0.86 goals, 95% CI (0.05, 1.67)], female teams demonstrated significantly higher values for *6-m centre* goals in an average match compared to male teams. Lastly, a significant effect of EHF EURO tournament was also found for *6-m centre* goals (large effect size), with values for an average match increasing from around four goals in the 2016 tournament to nearly eight goals in the 2024 tournament (see Tables 3, 4).

## Discussion

The primary aim of the current study was to examine how attacking play and goal-scoring patterns have evolved in elite men's and women's team handball over the past decade and explore how these trends can inform the design of representative training environments. Drawing on a comprehensive dataset comprising 666 matches from 10 EHF EURO championships (five men's and five women's tournaments) conducted between 2016 and 2024, the analysis focused on the distribution of goal types, spatial characteristics of scoring, and gender-based differences in offensive performance. The results revealed several notable patterns. Descriptively, organised positional attacks accounted for the largest share of goals across all championships and men and women, with later tournaments characterised by a higher proportion of close-range scoring. In the mixed-model analyses, tournament-related effects were most evident for 6-m centre goals, whereas other attack categories showed comparatively stable estimates across championships. Gender effects were observed for selected structured scoring categories (e.g., wing, 9-, and 6-m centre goals), while transitional and numerical imbalance situations showed limited gender differentiation.

As indicated in Tables 3, 4, there were clear descriptive patterns in attacking play across the EURO championships over the last decade. In both men's and women's competitions, positional attacks accounted for the majority of scoring actions, and their descriptive contribution increased between 2016 and 2024. For men’s teams, goals from positional attacks increased by approximately 2.5 per match across the period, while women's teams showed a comparable increase of around three goals per match. These descriptive patterns suggest an increasing reliance on structured offensive sequences and coordinated build-up play in generating goals against well-organised defensive systems. This interpretation is in line with previous evidence showing a progressive shift towards more structured offensive play in men's handball between 2012 and 2022 (11). By contrast, fast-break scoring remained descriptively stable or showed a slight reduction across tournaments. Men’s teams averaged approximately 1 less fast-break goal per match from 2016 to 2024, while women's teams showed an average reduction of around 0.5 goals per match over the same period. Despite their high scoring efficiency, the lower descriptive frequency of fast breaks may reflect improved defensive transition organisation, including faster return runs and better collective spatial recovery. This pattern aligns with earlier work suggesting that elite teams increasingly emphasise structured attacking play rather than relying on opportunistic transitions (11, 13).

Attacks under numerical superiority (majority play) and numerical inferiority (minority play) remained relatively stable across championships for both men’s and women’s teams, highlighting that while these situations are tactically significant, they account for a smaller share of overall scoring opportunities. Importantly, gender differences in majority-play scoring were minimal, reinforcing the notion that numerical advantages afford universal scoring opportunities regardless of physical or technical differences. Taken together, the distribution of goals across attack types suggests a broader tactical recalibration within top-performing EURO championship handball. Within these contexts, positional play and second-wave counterattacks constituted a substantial share of scoring actions, often associated with breakthrough situations and the creation of high-quality scoring opportunities against semi-structured defences. These trends reflect the adaptive nature of offensive strategies in response to improved defensive organisation, higher transitional discipline, and a general emphasis on reducing offensive risk. While the observed trends are consistent with evolving tactical and structural characteristics of elite championship play, alternative explanations should also be considered. Changes in competition rules, opponent quality and tactical variation, match dynamics and referee interpretation, goalkeeper development, athlete physical profiles, team composition, and defensive system organisation across tournaments may all contribute to the observed patterns. The present analysis cannot isolate the relative contribution of these factors, and the interpretations should therefore be understood as multi-factor performance associations rather than single-cause explanations.

The spatial distribution of goals is consistent with a tactical evolution in elite handball, with the 6-m zone emerging as the dominant scoring area. Goals from close range markedly increased across both men’s and women’s teams, highlighting the centrality of pivot play and penetrative actions in creating high-probability opportunities. Men's teams had, on average, 4.5 more 6-m goals per match in 2024 compared to 2016. In the same period, women's teams scored on average 3.2 goals more per match in the 6-m zone. Although both men's and women's teams demonstrated clear increases in 6-m scoring across the study period, men consistently exhibited higher values overall. Nevertheless, the parallel upward trends across genders underscore an increasing tactical emphasis on coordinated offensive structures designed to collapse defensive formations and exploit spaces around the goal area. Similar findings have been reported in earlier analyses of men's handball (11), suggesting a long-term trajectory towards proximal close-range finishing zones.

Wing shots followed a more variable trajectory across tournaments. In women's competitions, wing scoring sharply increased between 2016 and 2022, peaking at more than seven goals per match, before declining in 2024. For men’s teams, the peak occurred earlier, between 2018 and 2020, and had dropped by 2024. There is a considerable difference in the number of wing goals between men and women. While any explanation of these gender differences remains speculative, they may be associated with differences in physical profiles, defensive coverage patterns, or role-specific tactical emphases across competitions. In addition, this decline in wing-scoring frequency may reflect improved defensive strategies against wing play and a concurrent tactical recalibration towards central penetrations and pivot utilisation. The temporary resurgence of wing scoring in women's competitions suggests that tactical exploitation of wide spaces is subject to cyclical fluctuation, influenced by broader adjustments in offensive and defensive strategies.

Nine-metre shooting exhibited an apparent decline between 2016 and 2024, which aligns with earlier findings of trends in male handball competitions (18). On average, men's long-range scoring fell to two goals per match, and that of women's teams decreased to 2.4 goals in the same period. While still necessary for breaking defensive compactness and positioning longitudinally, long-range shooting now appears less central to offensive strategy. These patterns may be associated with improved goalkeeper anticipation and more aggressive perimeter defence, alongside a tactical preference for higher-probability, close-range finishing. Breakthrough goals—typically originating from passing the defender and jumping through the 6-m zone—substantially increased across men’s and women’s teams during the investigated period. Women's teams rose by an average of two goals per match from 2016 to 2024, while men's teams also rose by two goals per match. This increase is consistent with a growing tactical emphasis on one-on-one duels and creative individual actions—such as feints, tempo changes, and isolations—to destabilise compact defensive structures and create close-range scoring opportunities. These findings reinforce earlier statistical analyses that emphasise deception and individual initiative as critical elements of elite offensive play (28). The spatial analysis reveals a tactical evolution trend for men and women in elite handball, marked by a clear shift towards more goals in central scoring zones, particularly the 6-m area, as the primary locus of offensive effectiveness. This aligns with broader patterns in invasion sports, where proximity to the goal increasingly defines strategic success.

By situating these empirical findings within the broader context of applied sport science and coaching, this analysis addresses how large-scale performance trends can inform the design of training environments that promote representativeness from competition to practice. Longitudinal data from the EHF EURO championships (2016–2024) among top-performing teams indicate an apparent increase in both offensive frequency and goal output for men's and women's teams. Notably, gender-specific differences emerged in structured shooting outcomes, with male players exhibiting a higher frequency of goals from positional attacks, 6-m finishes, wing shots, and 9-m shots. In contrast, no significant gender differences were observed in transitional scoring situations, such as fast breaks or numerical imbalances. Moreover, tournament trends indicate a rise in goal frequency from positional attack settings, while the rate of counterattack goals remained relatively stable. Taken together, these associations suggest that, within comparable elite contexts, teams may increasingly need to practise high-quality, close-range scoring opportunities within complex, organised positional attacks.

In top-performing handball, skilled performance can be understood as being rooted in the players' ability to regulate their actions through the direct perception of dynamical affordances. These affordances are relational and ephemeral, shaped by the interplay between the players' capabilities and the game's spatiotemporal structure. Consequently, representative training should move beyond prescriptive, isolated repetition and instead incorporate open, information-rich tasks that demand real-time adaptation under pressure in attack. From an ecological dynamics perspective, attacking and defending are co-adaptive behaviours in which players continuously shape each other's possibilities for action (29). Representative attacking practice should therefore aim to replicate the informational complexity of competition, enabling players to attune to key variables such as defensive alignment, goal angle, time-to-contact, and fluctuating openings in passing, breakthrough, and shooting lanes. For backcourt players, who frequently initiate offensive structures, perceptual attunement to rates of change in spatial configurations appears critical for effective timing and decision-making.

Findings from the EURO data reinforce these theoretical insights concerning representativeness. However, the findings also suggest substantial variation in the positional localisation of goals, highlighting that attack training may therefore benefit from engaging involvement across the width of the positional attack. Wing players play a crucial role in creating defensive stretches; credible scoring threats from wide positions illustrate how local openings can trigger cascading affordances across positions. Accordingly, training designs can incorporate dynamic scenarios that destabilise defenders by encouraging lateral commitment, manipulating spacing, or altering positional configurations to reveal high-value opportunities.

Representative learning design (24) provides a theoretical approach for embedding affordance-rich scenarios into training. Through constraint manipulations—such as altering time pressure, changing defender start positions, or limiting passing options—coaches can encourage behaviours that reflect match-relevant decision thresholds. These conditions support players' perception of nested affordances, for example, a feint that opens a passing or breakthrough lane, which subsequently creates a close-range scoring opportunity (12). Passing requires particular attention to shared affordances (30), which emerge only when perceptual and temporal coordination between teammates is precisely aligned. Misalignment often results in turnovers or missed opportunities. Attack training may therefore benefit from supporting role synergy—the co-regulation of timing, space entry, and action selection—not as scripted patterns but as emergent behaviours shaped by realistic, constraint-rich contexts facilitated by the coach. Anchoring practice design in both ecological theory and match-derived patterns enables coaches to foster decision-making that is robust, flexible, and competition-ready (31–33).

Practically, representative training tasks should be designed to reflect the tactical and positional demands highlighted by the data. Given the increased scoring from structured attacks, exercises may simulate an overload in the temporal and spatial constraints of six-versus-six play. Backcourt players may be exposed to scenarios affording long-range shots, breakthrough actions, and coordinated penetrations into the 6-m zone. For wing players, training may extend beyond isolated finishing drills to include decision-making under pressure within the flow of positional attacks, forcing choices between wide-angle finishes and inside cuts. Similarly, for line players, whose scoring contributions have markedly risen, training may emphasise cooperative sequences with backcourt players, allowing interactions to emerge organically. These settings can promote attunement to spacing, timing, and defender positioning in ways that closely mirror competitive play.

Coaches are thus encouraged to critically evaluate traditional training paradigms, prioritising relational-action learning and systematically manipulating constraints in ways that simulate the perceptual complexity of match conditions and goal distributions revealed in the present trend analysis. Large-scale performance trends can provide a valuable resource for anticipatory coaching. By identifying relatively stable scoring patterns—such as the growing reliance on positional attacks and the stabilisation of counterattack efficiency—coaches can recalibrate tactical emphasis and session design.

### Limitations

Several limitations should be considered when interpreting the present findings. First, the sample was restricted to top-performing teams that progressed beyond the preliminary stages of the EHF EURO championships. While this improves comparability of competitive exposure, it limits the generalisability to broader performance levels and developmental contexts. Second, the analysis relied on secondary match statistics obtained from the official EHF data provider. Although these data are collected using standardised coding protocols, the study design does not allow independent verification of inter-rater reliability of event classification at the coding level. Third, the analytical model did not explicitly control for potentially influential contextual factors, such as opponent variation, tactical match-ups, roster variation, phase-specific game states, or differences in defensive systems. Consequently, the reported trends should be interpreted as structured performance pattern associations within elite championship contexts rather than as isolated causal effects. Fourth, changes in tournament formats across the study period—most notably the expansion of the women's EHF EURO in 2024—may confound tournament-year effects, as observed differences may partly reflect structural competition changes rather than tactical evolution alone.

## Conclusion

Across five EHF EURO cycles (2016–2024), patterns observed among top-performing teams indicate an increased emphasis on organised, centrally focused attacking. Positional attacks accounted for a growing share of goals, with marked rises in 6-m and breakthrough finishes, while fast-break scoring remained stable or slightly declined. These trends were evident in men and women, although men exhibited higher outputs in several structured categories (positional, wing, 6- and 9-m), with no meaningful gender gaps in transitional or numerical imbalance situations. Within comparable elite contexts, grounding practice in these empirical regularities suggests that coaches should prioritise representative six-versus-six tasks that preserve perceptual–temporal information, emphasise backcourt-to-pivot coordination and one-on-one creation, and maintain credible wing and long-range threats to stretch compact defences. Although the findings must be interpreted within the limitations of secondary match statistics and unmodelled contextual factors, the longitudinal trends provide a robust basis for evidence-informed representativeness in practice design.

Future research should examine whether these attacking trends generalise across other international competitions and competitive levels, particularly in contexts characterised by greater variation in opponent strength. In addition, deeper fine-grained observational analyses and the application of artificial intelligence may help identify stable information sources, affordance structures, and performer–environment interactions that underpin representative learning design in elite handball.

## Data Availability

Publicly available datasets were analysed in this study. This data can be found here: https://www.eurohandball.com.
