# Supplementary material for: Attacking and goal-scoring trends among top teams in EHF EURO handball (2016–2024): implications for representative practice design
Source: Front Sports Act Living. 2026 Mar 16;8:1771752. doi: 10.3389/fspor.2026.1771752 (PMC13033628; doi:10.3389/fspor.2026.1771752)
Supplement: Supplementary file 1 [file Table1.docx]

**Table S1.** Mapping of Offensive Performance Variables to Swiss Timing Definitions

| **Variable used in study** | **Swiss Timing label** | **Operational definition (Swiss Timing)** | **Notes on stability (2016–2024)** |
| --- | --- | --- | --- |
| **Attacks** | **Att** | **Discrete offensive team actions in ball possession, ending in a shot attempt, turnover, or loss of possession.** | **Definition and coding protocol unchanged across EHF EURO championships** |
| **Positional Attacks** | **Att (positional context)** | **Attacks executed against a fully organised defence, with ≥4 defenders set in defensive positions.** | **Stable definition; contextual classification applied consistently** |
| **Fast Breaks** | **FB** | **All shot attempts occurring before the defence is organised (i.e., before ≥4 defenders are set and ready to defend).** | **Stable definition** |
| **Team Fast Breaks** | **Team FB** | **Fast breaks involving more than one attacking player and at least one pass.** | **Stable definition** |
| **Individual Fast Breaks** | **Ind. FB** | **Fast breaks executed by a single player, typically following a steal or interception.** | **Stable definition** |
| **Fast Throw-Off** | **FTO** | **Fast attacks immediately following an opponent’s goal, with a maximum of three passes after throw-off.** | **Stable definition** |
| **Empty Goal Attacks** | **EG** | **Attacks executed while the defending team has no goalkeeper inside the six-metre area at the moment of the shot.** | **Stable definition** |
| **Majority Attacks** | **Att (numerical advantage)** | **Attacks executed while the opponent has one or more players temporarily suspended.** | **Stable definition** |
| **Minority Attacks** | **Att (numerical disadvantage)** | **Attacks executed while the attacking team has one or more players temporarily suspended.** | **Stable definition** |
| **Goals** | **G** | **All goals scored during match or tournament play (excluding 7m penalties when specified).** | **Stable definition** |
| **Shots** | **S** | **All throwing attempts directed towards the goal (goal, save, block, post, miss), excluding 7m penalties and free throws.** | **Stable definition** |
| **Nine-Metre Shots** | **9m** | **Shots taken from outside the nine-metre line (left/centre/right).** | **Stable definition** |
| **Six-Metre Centre Shots** | **6m** | **Shots taken at the six-metre line or from within the six-metre area, with no defender positioned between shooter and goal at execution.** | **Stable definition** |
| **Wing Shots** | **Wing** | **Shots taken within a 45° angle from the left or right wing, without a defender directly in front of the shooter.** | **Stable definition** |
| **Breakthrough Shots (1-on-1)** | **BT** | **Shots taken after a 1-on-1 breakthrough in the nine-metre zone, following an individual feint or dribble, without defensive assistance.** | **Stable definition; typically assigned to backcourt players** |
